# Visual Function After Schlemm’s Canal-Based MIGS

**DOI:** 10.3390/jcm14072531

**Published:** 2025-04-07

**Authors:** Masayuki Kasahara, Nobuyuki Shoji

**Affiliations:** Department of Ophthalmology, Kitasato University School of Medicine, 1-15-1 Kitasato, Minami-ku, Sagamihara 252-0374, Kanagawa, Japan; nobu_s@mua.biglobe.ne.jp

**Keywords:** minimally invasive glaucoma surgery (MIGS), Schlemm’s canal-opening surgery, trabeculotomy, canaloplasty, Kahook Dual Blade, trabectome, tanito microhook, visual acuity, visual field, astigmatism

## Abstract

Filtration surgery is highly effective in lowering intraocular pressure; however, it is associated with a higher risk of severe complications. Visual dysfunction may persist in relatively uneventful cases because of induced astigmatism or worsening optical aberrations. Therefore, for early- to moderate-stage glaucoma, an increasing number of surgeons are prioritizing surgical safety and preserving postoperative visual function by opting for minimally invasive glaucoma surgery (MIGS). Among the various MIGS techniques, canal-opening surgery—targeting aqueous outflow through the Schlemm’s canal (Schlemm’s canal-based MIGS, CB-MIGS)—has gained increasing popularity. Unlike filtration surgery, CB-MIGS does not require creating an aqueous outflow pathway between the intraocular and extraocular spaces. Consequently, it is considered a minimally invasive procedure with a reduced risk of severe complications and is increasingly being chosen for suitable cases. Although this surgical technique has limitations in lowering intraocular pressure, it avoids the manipulation of the conjunctiva or sclera and is primarily performed through a small corneal incision. Therefore, a minimal impact on induced astigmatism or postoperative refractive changes is expected. However, few reviews comprehensively summarize postoperative changes in visual function. Therefore, this study reviews the literature on visual function after CB-MIGS, focusing on changes in best-corrected visual acuity (BCVA), refraction, astigmatism, and the effectiveness of visual field preservation to assess the extent of these postoperative changes. Hyphema is the primary cause of early postoperative vision loss and is often transient in cases in which other complications would have led to visual impairment. Severe complications that threaten vision are rare. Additionally, compared with filtration surgery, postoperative visual recovery tends to be faster, and the degree of induced astigmatism is comparable to that of standalone cataract surgery. When combined with cataract surgery, the refractive error is at the same level as that of cataract surgery alone. However, in some cases, mild hyperopic shifts may occur because of axial length shortening, depending on the extent of intraocular pressure reduction. This possibility has been highlighted in several studies. Regarding the effectiveness of slowing the progression of visual field defects, most studies have focused on short- to medium-term postoperative outcomes. Many of these studies have reported the sufficient suppression of progression rates. However, studies with large sample sizes and long-term prospective designs are limited. To establish more robust evidence, future research should focus on conducting larger-scale, long-term investigations.

## 1. Introduction

Glaucoma is the leading cause of blindness worldwide [1]. As the global population continues to age, the number of patients with glaucoma is projected to increase from 76 million in 2020 to 95.4 million by 2030 [2]. Elevated intraocular pressure (IOP) is a major risk factor for disease onset and progression [3,4], and lowering the IOP slows disease progression [5,6]. First-line treatments for reducing IOP include topical medications and selective laser trabeculoplasty [7,8]. However, when these conservative treatments fail to provide long-term control or prevent disease progression, glaucoma surgery should be considered. Glaucoma surgery aimed to lower IOP in a safe and sustained manner over the long term. Although filtration surgery is highly effective in reducing IOP, it is associated with a high risk of severe complications. Even in patients with an uneventful postoperative course, a long recovery period is often required to restore visual acuity. In addition, refractive changes, increased astigmatism, and the exacerbation of optical aberrations may result in reduced unaided visual acuity or persistent visual impairment [9,10].

Therefore, in early- to moderate-stage glaucoma, surgical safety, the speed of postoperative visual recovery, and the preservation of long-term visual function are often prioritized. Consequently, minimally invasive glaucoma surgery (MIGS) has been increasingly used in such cases. Among the MIGS techniques, canal-opening surgery, which targets aqueous outflow through the Schlemm’s canal, is classified as Schlemm’s canal-based MIGS (CB-MIGS). Depending on the mechanism used to enhance aqueous outflow through the main drainage pathway, CB-MIGS can be further categorized into several surgical methods, each utilizing different devices.

A common feature of these device-based procedures is that they are minimally invasive, have a lower risk of severe complications [11,12,13], are relatively simple and quick to perform, and do not typically require conjunctival incisions, thereby preserving the possibility of future filtration surgery. Due to their mechanism of action, CB-MIGS cannot lower IOP beyond episcleral venous pressure. However, unlike filtration surgery, CB-MIGS rarely results in excessively low single-digit IOP. Moreover, because it is primarily performed through small corneal incisions without manipulating the conjunctiva or sclera, a minimal impact on induced astigmatism or postoperative refractive changes is expected.

Another important focus is how effectively CB-MIGS can slow the postoperative progression of visual field defects, although it generally achieves less IOP reduction than filtration surgery. While numerous reports and reviews have examined the IOP-lowering effects and complications of CB-MIGS, relatively few comprehensive reviews have detailed the changes in visual function, such as best-corrected visual acuity (BCVA), refraction, astigmatism, or maintenance of visual fields after surgery.

This review summarizes the existing literature on visual function outcomes after CB-MIGS. Two authors (M.K. and N.S.) independently conducted the literature search in October 2024. The search was performed using PubMed and targeted full-text articles or abstracts written in English. Articles published in peer-reviewed journals over the past 20 years were selected if they were relevant to the topic of this review and contributed to a deeper understanding of the visual function after CB-MIGS. Relevant studies cited in the references of the retrieved articles were also included. In cases where the authors had differing opinions on the relevance of a study, a disputed study was included.

## 2. Types and Characteristics of CB-MIGS

CB-MIGS can be broadly classified into three categories based on their underlying principles. Ab interno trabeculotomy (AIT), a procedure involving incising or excising the trabecular meshwork, bypassing the trabecular meshwork using implantable devices, which involves placing stents to bypass the anterior chamber and Schlemm’s canal, and Ab interno canaloplasty (ABiC) is a procedure performed ab interno to expand the Schlemm’s canal without incising or excising the trabecular meshwork.

### 2.1. AIT

The Trabectome^®^, introduced by Minckler et al. in 2005 [14], represents the first device specifically designed to internally ablate the trabecular meshwork, marking a significant milestone in MIGS development. The device features a handpiece equipped with a 19-gauge infusion sleeve, a 25-gauge aspiration port, and a moderately sharp triangular tip. The inner wall of the trabecular meshwork was ablated using a mechanism resembling bipolar cautery [14].

Subsequently, the Kahook Dual Blade (KDB) was developed as an alternative. Although it does not include a cauterization function, the KDB does not require expensive equipment and allows the excision of the trabecular meshwork using its dual-bladed tip. Numerous studies have demonstrated the safety and efficacy of both the Trabectome (TOM) [15,16,17,18,19,20,21,22,23,24,25,26,27,28,29,30,31,32,33,34,35,36,37,38,39,40,41,42,43,44,45,46,47,48,49,50,51,52,53,54,55] and KDB procedures [56,57,58,59,60,61,62,63,64,65,66,67,68,69,70,71,72,73,74,75,76,77,78,79,80,81,82,83,84,85,86].

The Tanito Microhook (TMH), developed to incise rather than excise the trabecular meshwork, differs from TOM and KDB because it is a reusable device, offering superior cost-effectiveness. The IOP-lowering effects of TMH are comparable to those of TOM, KDB, and trabeculotomy ab externo (exLOT) [47,87,88,89,90,91,92,93,94,95,96,97,98,99].

In 2014, Grover introduced gonioscopy-assisted transluminal trabeculotomy (GATT), a procedure that uses a microcatheter or suture to achieve a 360-degree incision in the trabecular meshwork ab interno. The safety and efficacy of GATT have been well established [100,101,102,103,104,105,106,107,108,109,110,111,112,113,114,115,116,117,118]. Regarding the extent of the incision, whether performing a 360-degree incision offers superior IOP reduction compared with a 90–150-degree incision remains unclear. Some studies suggest that a larger incision area results in greater IOP reduction [106,119,120,121], whereas others report no significant difference in IOP reduction beyond a 90-degree incision [97,122,123,124,125,126,127]. This is currently an area of ongoing research.

### 2.2. Bypassing the Trabecular Meshwork Using Implantable Devices

The iStent (Glaukos Corporation, San Clemente, CA, USA) and Hydrus (Alcon Laboratories, FortWorth, TX, USA) are the most widely used micro-implantable devices in the market. The first-generation design was a single nonferromagnetic titanium stent with an inlet attached at a 40-degree angle to an intracanalicular half-cylinder coated with heparin to prevent fibrosis [128]. The second-generation iStent injection comprises two button-shaped titanium stents delivered through an updated delivery system. The stents feature a head that resides in the Schlemm’s canal, a thorax that anchors in the trabecular meshwork, and a rear flange that resides in the anterior chamber. Third-generation iStent Injection W represents an iterative improvement, featuring a wider rear flange designed to reduce the risk of overimplantation. The iStent, which does not involve incising the trabecular meshwork, is minimally invasive and effective [129,130,131,132,133,134]. However, its efficacy may be lower compared with AIT [135,136,137].

In contrast, the Hydrus, a Schlemm’s canal scaffold, combines elements of canaloplasty and the iStent to dilate Schlemm’s canal and establish a connection between the anterior chamber and Schlemm’s canal [138]. The stent is made of a nonferromagnetic nickel–titanium alloy and features a curved, flexible, open structure with windows and spines designed to dilate approximately 8 mm of the Schlemm’s canal to four to five times its physiological diameter. Moreover, its efficacy and safety have been well documented [138,139,140,141,142].

### 2.3. ABiC

Rather than creating a wide incision in the Schlemm’s canal, canaloplasty facilitates aqueous outflow by expanding the canal and has gained widespread adoption, particularly in Europe. The procedure involves the viscodilation of the Schlemm’s canal, the insertion of a microcatheter, and the stretching of the trabecular meshwork using a 10-0 prolene thread, thereby improving aqueous outflow.

Traditional canaloplasty is performed externally and is not classified as MIGS. However, more recently, ABiC, which uses specific devices, such as iTrack (Ellex Inc., Minneapolis, MN, USA) and OMNI (Sight Sciences Inc, Menlo Park, CA, USA), has been considered a form of MIGS. Numerous studies have reported their safety and efficacy [115,138,143,144,145,146,147,148,149,150,151,152,153,154,155,156,157,158,159,160,161]. Additionally, OMNI enables AIT to be performed after canaloplasty [162,163,164,165,166,167].

A common feature of CB-MIGS is that these procedures are performed through a small corneal incision using the ab interno approach. These outflow reconstruction surgeries redirect the aqueous humor to the collector channels and episcleral veins. Consequently, CB-MIGS can lower the IOP; however, it cannot reduce it below the episcleral venous pressure (8–10 mmHg) or the resistance levels of the distal outflow pathways. In contrast, CB-MIGS rarely causes severe complications associated with filtration surgeries, such as hypotony-related issues, infections, or expulsive hemorrhages. This positions CB-MIGS as a safe and reliable surgical option [11,12,13,14,15,16,17,18,19,20,21,22,23,24,25,26,27,28,29,30,31,32,33,34,35,36,37,38,39,40,41,42,43,44,45,46,47,48,49,50,51,52,53,54,55,56,57,58,59,60,61,62,63,64,65,66,67,68,69,70,71,72,73,74,75,76,77,78,79,80,81,82,83,84,85,86,87,88,89,90,91,92,93,94,95,96,97,98,99,100,101,102,103,104,105,106,107,108,109,110,111,112,113,114,115,116,117,118,119,120,121,122,123,124,125,126,127,128,129,130,131,132,133,134,135,136,137,138,139,140,141,142,143,144,145,146,147,148,149,150,151,152,153,154,155,156,157,158,159,160,161,162,163,164,165,166,167].

## 3. Surgical Indications for CB-MIGS

It is essential to observe the anterior chamber angle intraoperatively, with a Shaffer grade between 2 and 4. Additionally, the trabecular meshwork at the incision or implant site should ideally be free of peripheral anterior synechia (PAS). However, in AIT, a small tent-shaped PAS can be released using the instrument tip during the incision. The target intraocular pressure (IOP) is in the mid-10 mmHg range, making cases with an IOP exceeding 20 mmHg ideal candidates. However, surgery may also be considered for patients with an IOP in the 10 mmHg range, depending on medication adherence, side effects, and IOP fluctuations. For example, surgery may be appropriate for elderly patients with dementia who struggle with eye drop adherence, those experiencing severe keratitis or blepharitis from eye drops, or individuals with systemic symptoms—such as fatigue, loss of appetite, or a history of urinary stones—that hinder acetazolamide therapy. Significant IOP fluctuations, particularly with nighttime elevation, may also indicate the need for surgery. Conversely, surgery is not actively recommended for patients who can manage eye drops and maintain an IOP in the mid-to-high 10 mmHg range, even if visual field defects continue to progress. In cases where the visual field defect is already in the late stage or beyond, filtration surgery may be a more suitable option.

## 4. Visual Acuity After CB-MIGS

Visual acuity often declines after trabeculectomy (TLE), and recovery can take considerable time. Moreover, some cases experience permanent visual impairment [9,10]. In a study conducted by the authors on visual changes following TLE in 292 eyes, 4.45% of the patients exhibited a decline in BCVA of two or more lines on the Snellen chart that did not recover by 3 months postoperatively [168]. Similarly, Francis et al. analyzed data on 301 eyes that underwent TLE and reported that 64.5% of cases experienced a decline in three or more Snellen chart lines within 6 months postoperatively. Of these, 56.5% recovered within 6 months, while 8.0% did not recover [169].

However, the proportion of cases in which BCVA declines by two or more Snellen chart lines after CB-MIGS is 0–5% for AIT [15,16,17,18,39,56,57,58,59,70,92,170,171,172,173,174,175], 0–9.7% for micro-implantable devices [139,140,141,142,176,177,178,179,180,181,182,183,184,185,186,187,188,189,190,191,192,193,194,195], and 1.2–3% for ABiC [146,148,196,197], which is lower than that of TLE. Additionally, many studies have reported no severe complications that significantly affect visual function.

Additionally, reports showing a ≥7% incidence of visual decline identified several causes. These included the progression of pre-existing cataracts during long-term follow-up after iStent implantation [183] and a transient two-line or greater decrease on the Snellen chart due to corneal edema from an IOP spike and hyphema on the day after Hydrus implantation [140]. Variations in observation time points, unclear reporting, and the inclusion of simultaneous cataract surgery make accurate result interpretation challenging.

When proposing CB-MIGS, it is crucial to understand whether the postoperative BCVA reduction is due to direct surgical complications and whether such reductions are temporary or permanent. A long-term decline in visual acuity may occur when the procedure is performed in advanced cases with severe preoperative sensitivity loss near the fixation point. Moreover, the progression of cataracts or the development of other diseases that affect visual acuity can contribute to a decline in BCVA. Therefore, reviews that merely quantify the percentage of BCVA reduction are limited. It is critical to first explain the extent, types, and frequency of complications that may influence visual acuity in the chronological order of their occurrence.

## 5. Visual Decline Due to Complications

### 5.1. Hyphema

Hyphema is the most common complication causing early postoperative visual decline after CB-MIGS. When the IOP temporarily drops below the episcleral venous pressure postoperatively, reflux bleeding can result in hyphema. In the absence of intraoperative iris trauma or damage to the angle vessels, postoperative hyphema serves as evidence that the outflow pathway beyond the collector channels has been connected to the episcleral veins. This is also considered a positive indicator of IOP reduction.

Hyphema is not limited to AIT or micro-implantable devices; it is also a common complication of ABiC, where the partial incision of the trabecular meshwork during the expansion of Schlemm’s canal can lead to bleeding. The reported incidence of hyphema varies depending on the procedure used. For iStent and iStent injections, the incidence ranged from 0 to 11.4%, whereas it ranged from 1.9 to 6.5% for Hydrus. Procedures, such as the KDB, have reported an incidence of 0–34.9%, whereas the TOM has a wider range of 4.7–95.0%. For TMH, the incidence is 14–41%, while that for GATT ranges from 2.0 to 100.0%. Similarly, the incidence of ABiC ranges from 1.0 to 47.0% (Table 1).

Visual acuity naturally reduces, whereas hyphema persists postoperatively. However, in most cases, bleeding resolves spontaneously within approximately 1 week, and visual acuity returns to baseline [34,59,68,71,78,80,103,115].

In rare cases, massive or prolonged bleeding may require anterior chamber irrigation because such conditions can lead to elevated IOP or adverse effects on the corneal endothelium. Reports indicate that anterior chamber irrigation was required in 0–9.0% of cases following AIT [21,84,88,92,93] and in 0–10.9% of cases following GATT [102,105,112,114,116,198]. However, no previous studies have identified this as a direct cause of permanent visual impairment. However, MIGS aims to promote early visual recovery, and bleeding should ideally be avoided or, if it occurs, should remain minimal and resolve spontaneously within a short period.

Regarding the speed of visual recovery in CB-MIGS alone, Tanito et al. reported postoperative visual outcomes in 159 eyes treated with TMH alone [92]. The preoperative logMAR BCVA was 0.11 ± 0.39. Postoperatively, the BCVA was 0.30 ± 0.68 at 1–2 weeks (*p* < 0.0001), 0.15 ± 0.48 at 1 month (*p* = 0.02), and 0.07 ± 0.29 at 3 months (*p* = 0.83). These results indicate that while visual acuity recovered to levels close to the preoperative values after 1 month, a statistically significant difference remained at that point. In contrast, reports on iStent and iStent injections suggest that, unlike AIT, these procedures do not require an incision of the trabecular meshwork. Consequently, early postoperative complications, including hyphema, are less frequent [130,133,134,137], and early postoperative visual recovery is often expected [133].

In a comparative study of TMH, Harano et al. evaluated 78 eyes of 39 patients who underwent simultaneous cataract surgery with TMH in one eye (TMH group) and iStent injection of W in the contralateral eye (injection W group) [136]. The incidence of hyphema in the injection of W group was 5%, which was significantly lower than that in the TMH group (26%; *p* = 0.025). Additionally, the BCVA in the injection of W group was significantly better at 2 weeks (*p* = 0.005) and 1 month (*p* < 0.0001) postoperatively, whereas no significant difference in BCVA was observed between the two groups at 3 months postoperatively.

These characteristics make them particularly suitable for patients requiring early postoperative visual recovery in order to facilitate a quick return to social and occupational activities.

The frequency and severity of hyphema vary not only between different surgical techniques but also considerably within the same technique across various reports. This discrepancy can be attributed to three main factors. First, the definition of hyphema has not been standardized. Although many studies define hyphema as the formation of a blood niveau in the anterior chamber [88,92,93], some reports, although not explicitly stated, may include coagulated blood clots in their definition. Furthermore, hyphema does not always present as blood niveau; it can also manifest as individual red blood cells floating in the anterior chamber. This flutter-type hyphema is challenging to quantify and is often omitted from reports. However, it is believed to have an impact on visual acuity reports. Sugihara et al. reported that when flutter-type hyphema was included, hyphema was observed in 97% of cases after TMH with a nasal incision alone and in 100% of cases after TMH with both nasal and temporal incisions [97].

Second, differences in the extent of the incision may influence the frequency and severity of hyphema. In procedures like TOM and KDB, it is common to incise or excise the trabecular meshwork over a range of approximately 90–120 degrees through a single corneal port [20,21,26,56,199]. In contrast, TMH with curved hooks allows for a broader incision range. Incisions may cover 90–240 degrees using one or two corneal ports [88,89,97,200,201], whereas GATT typically involves a 360-degree incision of the trabecular meshwork [100,101,105,112].

Reports of hyphema persisting beyond 1 week postoperatively or even beyond 1 month, requiring additional anterior chamber irrigation, are more prominent in studies involving 360-degree incisions than in those involving 120-degree incisions [102,105,112,114,116]. However, reports of anterior chamber irrigation have also been noted in studies on TOM, TMH, and KDB [21,84,88,92,93], making it challenging to compare techniques based on retrospective studies.

Several studies have compared the incidence of hyphema based on different incision ranges when using TMH for AIT. Okada et al. compared the 120-degree and 180-degree incisions [200], whereas Mori et al. compared the 120-degree and 240-degree incisions [201]. In all these studies, no significant differences in IOP reduction were observed between the groups. However, broader incision ranges were associated with a higher incidence of hyphema [97,200,201]. It is important to consider the potential for delayed visual recovery when choosing surgical techniques involving extensive TM incisions.

Third, differences in IOP settings at the time of wound closure may influence the degree of reflux bleeding from the episcleral veins. Although no studies have directly compared the incidence of hyphema based on IOP levels at closure, the difference in hyphema incidence between ab externo and ab interno trabeculotomy techniques may provide some insights.

Kanda et al. compared the incidence of hyphema between AIT and exLOT and found that hyphema occurred in 33% of cases with exLOT and 11% with AIT, with the incidence being significantly lower in AIT (*p* < 0.01) [96]. The higher incidence of hyphema in exLOT may be attributed to the use of a sinusotomy, which facilitates aqueous humor filtration into the external environment, making achieving a low IOP more likely and predisposing patients to reflux bleeding. By contrast, AIT may reduce the likelihood of reflux bleeding from the episcleral veins by setting a higher IOP during hydrosealing at wound closure.

As IOP settings at the time of closure vary, depending on the surgeon’s preferences and individual cases, this factor may contribute to differences in the incidence of hyphema in CB-MIGS. To reduce the frequency of hyphema, the surgeons routinely aimed to complete a procedure with relatively higher IOP settings at the time of wound closure.

In GATT combined with cataract surgery, Loayza-Gamboa et al. reported a low incidence of hyphema (9.3%) [202], whereas Cubuk et al. reported a much higher incidence (89.1%) [203]. Cubuk et al. attributed the high frequency of hyphema to the omission of filling the anterior chamber with viscoelastic material at the end of the procedure [203], suggesting that this step may help prevent postoperative hyphema [100]. Additionally, recent studies have identified exfoliation glaucoma (XFG) as a risk factor for increased hyphema incidence [84,204,205]. Many patients with glaucoma are elderly and often receive anticoagulants, while some studies have found no association between anticoagulant and hyphema incidence [84,205,206], Chihara et al., in their study of exLOT, reported a significant difference in hyphema incidence between patients who discontinued the anticoagulant therapy and those who did not. They further noted that patients who continued anticoagulant therapy experienced prolonged hyphema, required more anterior chamber irrigation, and showed differences in early safety and complications compared with those who discontinued the therapy [207].

However, they also reported no significant difference in IOP 1 year postoperatively between the two groups. Given the potential risk of ischemic systemic diseases such as stroke or myocardial infarction, which could affect life expectancy, the decision to discontinue anticoagulant therapy remains unclear.

### 5.2. IOP Spikes and Corneal Edema

In addition to hyphema, corneal edema is another postoperative complication that can affect early visual recovery [29,56,68,70,75,104]. Baykara et al. reported transient corneal edema in five eyes (6.2%) in their study on the outcomes of GATT using TRAB360 (Sight Sciences Inc, Menlo Park, CA, USA) in 81 eyes with primary open-angle glaucoma (POAG) [105]. Corneal edema can occur when surgery is prolonged because of technical challenges, in cases with pre-existing endothelial dysfunction, or because of postoperative IOP spikes. Sakamoto et al. identified long axial length as a risk factor for IOP spikes [85]. The reported incidence of IOP spikes varies by procedure, with rates of 1.0–28.9% for AIT [15,16,17,18,39,56,57,58,59,70,88,92,93,95,170,171,172,173,174,175], 0–49.0% for GATT [100,104,105,113,202,208,209,210,211,212], 1.1–22.2% for iStent and iStent inject [176,177,178,179,180,181,182,183,184,185,186,187,188,189,190,191,192,193,194], 1.9–6.5% for Hydrus [139,140,141,142,195], and 0–42.0% for ABiC [146,148,196,197]. In most cases, IOP spikes are transient and can be managed by adding IOP-lowering medications. However, in advanced cases with preoperative sensitivity loss extending near the fixation point, such spikes may exacerbate the sensitivity loss and potentially lead to permanent visual impairment. Therefore, careful consideration is necessary when determining surgical indications for such patients [52].

### 5.3. Cystic Macular Edema (CME)

CME is a frequently observed complication, with a reported incidence of 1.8–7.0% [31,39,55,57,65,68,69,70,81,86,88,93,107,213]. While some cases of CME are triggered by intraoperative inflammation, others are induced by pre-existing ischemic retinal conditions such as retinal vein occlusion or inflammatory diseases. Most cases are temporary and improve with treatment.

Luebke et al. reported clinically significant postoperative CME (defined as CME associated with visual impairment or confirmed by optical coherence tomography) in three (2.2%) of 137 eyes that underwent TOM combined with cataract surgery [213]. Of these cases, two eyes were diagnosed with exfoliation glaucoma, and one eye had primary open-angle glaucoma. Local corticosteroid therapy, topical non-steroidal anti-inflammatory agents, and systemic acetazolamide were administered for 6 weeks, resulting in the improvement of both CME and visual acuity in all three eyes.

In contrast, the study examined a control group of 1704 eyes that underwent standalone cataract surgery and reported a 1.9% incidence of CME in this group. Cataract surgery alone has been previously associated with a 20–30% incidence of CME, as detected by angiography [214], with clinically significant CME affecting visual acuity reported in 1–2.4% of cases [215,216]. These findings suggest that the cataract procedure may influence CME incidence in CB-MIGS combined with cataract surgery. In previous reports that specified whether CME occurred after standalone CB-MIGS or simultaneous cataract surgery, most cases were associated with cataract surgery [31,65,68,69,81,86,88,213].

In our study, 2 out of 46 eyes that underwent standalone AIT developed CME [55]. One case was linked to central retinal vein occlusion (CRVO), which occurred incidentally two months postoperatively, while the cause in the other case remained unclear. However, after a subtenon triamcinolone acetonide injection, visual acuity recovered to baseline. Additionally, a separate report documented one case of CME among 53 eyes that underwent standalone KDB, though the cause was unspecified [57]. Another study reported one case of CME among 22 standalone KDB cases, where the patient had a history of multiple intraocular surgeries and eventually developed uncontrollable iritis, presumed to be the cause of CME. Although the incidence of CME following standalone CB-MIGS appears to be much lower than with simultaneous cataract surgery, it remains a potential complication.

### 5.4. Complications Potentially Leading to Severe Visual Impairment

Although severe and permanent visual loss due to CB-MIGS complications is extremely rare, it can still occur. Kaplowitz et al. reported that [217] the incidence of hypotony < 5 mmHg after TOM was 0.09% [174,218]. Most cases are believed to result from intraoperative errors, leading to angle disinsertion. In cases where macular folds persist, treatment options, such as the injection of viscoelastic agents into the anterior chamber or laser therapy, may be considered [219].

In addition, the risk of endophthalmitis, a complication often associated with filtration surgery, is extremely low in CB-MIGS performed using small corneal incisions. One reported case of postoperative endophthalmitis caused by *Enterococcus faecalis* was detected through culture, with a meta-analysis indicating an incidence of 0.01% [220]. However, at our facility, we experienced one case of endophthalmitis [18]: a patient with severe atopic dermatitis, associated lagophthalmos, and a history of multiple intraocular surgeries. Fortunately, following vitrectomy and antibiotic therapy, the patient’s vision was recovered, and the IOP was well controlled.

Patients with atopic dermatitis are highly likely to harbor resistant bacteria, such as methicillin-resistant *Staphylococcus aureus* or methicillin-resistant *Staphylococcus epidermidis,* in their conjunctival sacs [221]. Therefore, careful monitoring for postoperative endophthalmitis is essential.

Similarly, suprachoroidal hemorrhage, another serious complication of filtration surgery, is exceedingly rare in CB-MIGS, with a reported incidence of 0.01% [222]. In a recent study, Waldner et al. compared the outcomes of superior and inferior hemi-section GATT in 297 eyes and reported two cases of suprachoroidal hemorrhage. However, both cases were rapidly resolved with appropriate management [114].

### 5.5. Delayed-Onset Hyphema

Delayed-onset hyphema is a complication that may occur > 2 months after surgery, often resulting in mild visual decline. Patients typically present with a sudden blurring of vision after an extended postoperative period. On slit-lamp examination, floating red blood cells (flutter-type hyphema) are often observed in the anterior chamber, and gonioscopy frequently reveals mild hyphema localized inferiorly.

To date, 12 cases of delayed-onset hyphema occurring > 2 months after TOM have been reported [223]. Other cases have also been documented, including one case occurring 6 months after GATT [100], one 13 months after iStent implantation [224], and one 4 years after TMH [225]. The proposed mechanisms for these events include an increase in episcleral venous pressure due to physical exertion or sudden decompression caused by ocular compression during sleep [223].

However, delayed-onset hyphema following CB-MIGS is generally mild and self-limiting. Management typically involves observation rather than aggressive intervention. If a transient mild increase in IOP is observed, the addition of IOP-lowering medications is recommended.

Given that the distal outflow pathways beyond the collector channels connected to the episcleral veins remain functional, recognizing the potential for delayed-onset hyphema, even in the long-term postoperative period, is essential.

### 5.6. Cataract Progression

Since CB-MIGS is an intraocular procedure, cataract progression leading to postoperative visual decline may occur if the crystalline lens is preserved during surgery [51,113,115,178]. Particular attention should be paid to uveitic glaucoma (UG) as it poses a higher risk of cataract progression than other types of glaucoma [113,226,227].

Kijima et al. conducted a study involving 51 eyes with UG and 51 eyes with POAG that underwent the GATT. They reported that in the UG group, 29% of cases required additional cataract surgery within 12 months postoperatively, which was significantly higher than the 1.9% in the POAG group (*p* < 0.01) [113].

When performing surgery alone for UG, informing patients about the potential risk of postoperative cataract progression is essential.

### 5.7. Reduction in Corneal Endothelial Cell Density (ECD)

A reduction in ECD is a potential complication that can lead to visual decline in the long-term postoperative period. Among prior reports, no CB-MIGS procedures exist aside from the CyPass supraciliary stent, which was withdrawn from the market owing to high postoperative ECD loss [228], raising significant concerns regarding substantial ECD reduction [19,88].

The authors conducted a study of 159 eyes to evaluate changes in ECD before and after TOM. The findings indicated no significant reduction in ECD over 3 years, even when measured in six peripheral directions, 3 mm from the central cornea [229]. Although procedures involving device implantation may raise concerns about accelerated ECD loss, a report by Hydrus indicated that, while surgical trauma may initially reduce ECD, the rate of ECD loss beyond 3 months postoperatively was not significantly different from that observed in standalone cataract surgery [228].

### 5.8. Glaucoma Progression

Although severe complications that threaten vision are rarely observed in advanced cases, there remains a significant possibility of visual decline due to glaucoma progression. Regarding long-term visual outcomes after CB-MIGS, Kitamura et al. studied 96 eyes treated with TOM alone and reported that the BCVA significantly decreased at 1 week postoperatively. However, no significant differences in BCVA were observed between the preoperative and postoperative periods from 2 weeks to 57 months [53].

Conversely, Un et al. evaluated 60 eyes that underwent TOM with a mean follow-up period of 59.4 ± 14.3 months. They reported a significant deterioration in BCVA, which worsened from a preoperative logMAR value of 0.41 ± 0.49 to 0.61 ± 0.75 at the final follow-up (*p* = 0.02). However, during follow-up, five eyes developed other ocular conditions, such as age-related macular degeneration, diabetic maculopathy, or branch retinal vein occlusion [51], leaving it unclear whether the visual decline was due to glaucoma progression alone. In a study involving 305 eyes with a follow-up period of approximately 72 months after TOM [18], a relatively high proportion of advanced cases were included, and 44% of the eyes ultimately required additional filtration surgery. Although some cases involved the re-elevation of IOP, many also exhibited progressive visual field loss or the worsening of visual acuity despite relatively good IOP control.

Notably, cases requiring additional surgery were excluded from subsequent analyses, which may have limited the ability of such studies to fully reflect the true course of visual outcomes following CB-MIGS. Moreover, several recent reports have raised concerns regarding the long-term efficacy of CB-MIGS [18,52]. This is particularly true in advanced cases with preoperative sensitivity loss near the fixation point, where the limited effectiveness of CB-MIGS renders it unsuitable. In such cases, CB-MIGS should be avoided as a treatment option [18,52,53].

## 6. Refractive Changes

Previous studies have highlighted that patients with glaucoma are at a higher risk of refractive shifts during cataract surgery [230,231], and the accuracy of intraocular lens (IOL) power calculation tends to be low in these patients [232,233]. Furthermore, in filtration surgery, it is common for the IOP to drop to single-digit levels, which results in postoperative axial length shortening [234].

Francis et al. conducted a study of 39 eyes that underwent TLE and 22 eyes with Baerveldt glaucoma implants. At 3 months postoperatively, the mean IOP reduction and axial length shortening were −12.8 ± 1.5 mmHg and −0.16 ± 0.03 mm in the TLE group, and −10.7 ± 1.9 mmHg and −0.15 ± 0.03 mm in the Baerveldt group, respectively. In both groups, the axial length was significantly shorter than the preoperative values (*p* < 0.001). Additionally, the extent of axial length shortening beyond 3 months postoperatively was associated with postoperative IOP levels and the magnitude of IOP reduction (*p* < 0.05) [235].

Regarding the refractive impact of filtration surgery, Cherecheanu et al. reported on 21 eyes that underwent TLE at least 6 months after cataract surgery. They observed a 0.40 ± 0.13% reduction in axial length and an average hyperopic shift of 0.34 ± 0.44 D at 12 months postoperatively (*p* = 0.002) [236]. During filtration surgery, in addition to axial length shortening due to IOP reduction, anatomical changes such as shifts in the effective lens position (ELP) and anterior chamber depth (ACD) may also occur. Therefore, the potential for postoperative refractive abnormalities should be carefully considered when performing combined cataract and filtration surgery [235,237,238,239].

In CB-MIGS, the reduction in IOP is not as dramatic as that observed with filtration surgery, and the impact on axial length and refractive changes is considered less significant. However, in cases with high preoperative IOP, some patients experience IOP reductions > 10 mmHg, which could potentially influence the axial length.

In a study involving 53 eyes, Tekcan et al. reported that the mean axial length was significantly shortened after GATT combined with cataract surgery [240]. Similarly, Kanda et al. conducted a detailed analysis of IOP reduction and axial length changes in 52 eyes that underwent TMH combined with cataract surgery (TMH group) and 67 eyes that underwent cataract surgery alone (cataract group) [241]. In the TMH group, preoperative IOP decreased from 19.0 ± 6.0 mmHg to 12.2 ± 2.3 mmHg at 1 month postoperatively (*p* < 0.001), and axial length shortened from 23.98 ± 1.17 mm preoperatively to 23.82 ± 1.16 mm postoperatively. In the cataract group, the preoperative IOP decreased from 14.7 ± 2.5 mmHg to 13.3 ± 2.6 mmHg at 1 month postoperatively (*p* = 0.002), while the axial length changed from 23.79 ± 1.06 mm preoperatively to 23.68 ± 1.08 mm postoperatively.

Although no statistically significant differences in axial length were observed between the group pre- and postoperatively, the TMH group exhibited a significantly greater reduction (*p* < 0.001) when the magnitude of axial length changes between the groups was compared. Furthermore, the extent of IOP reduction was correlated with the degree of axial length shortening (*p* = 0.01) [241].

In addition to axial length, anatomical factors, such as ELP and ACD, can potentially affect refractive changes. However, CB-MIGS reduces IOP by bypassing the trabecular meshwork, causing minimal alterations to the surrounding structures. This contrasts with filtration surgery, in which significant anatomical changes are more likely. Consequently, CB-MIGS impacts on the target refractive error should be minimal, even in combined cataract surgery [242].

## 7. Recent Advances in Target Refractive Error in Cataract Surgery

The target refractive error in standalone cataract surgery has steadily decreased in recent years owing to advancements in diagnostic devices and IOL power calculation [243,244,245,246,247,248,249]. Reports from 1992 to 2006 indicated that the percentage of eyes achieving a target refractive error within ±1.0 D is 72.3–87.0% [243,244,245,246]. However, between 2007 and 2017, it increased to 89.6–97.3% [247,248,249]. Regarding combined CB-MIGS and cataract surgery, Scott et al. compared 76 eyes that underwent iStent implantation combined with cataract surgery with 50 eyes that underwent cataract surgery alone. They reported that the percentage of eyes achieving a target refractive error within ±0.50 D was 80% in both groups, while the percentage within ±1.0 D was 95% and 94%, respectively, with no significant difference between the groups (*p* > 0.05) [250] (Table 2). Rho et al. examined 36 eyes that underwent iStent inject implantation combined with cataract surgery and found an absolute predicted error (APE) of 0.33 ± 0.26 D, with 83.3% achieving a target refractive error within ±0.50 D. This was comparable to a control group of 100 eyes that underwent standalone cataract surgery, with no significant differences between the groups [251]. Similarly, Ioannidis et al. evaluated 106 eyes that underwent iStent inject implantation (Glaukos Corporation, San Clemente, CA, USA) combined with cataract surgery and reported an APE of 0.36 ± 0.25 D. In this study, 73.9% of eyes achieved a target refractive error within ±0.50 D, and 98.9% were within ±1.0 D [252]. Furthermore, no significant correlation was observed between preoperative IOP or IOP fluctuation and the postoperative refractive target (*p* > 0.05). The refractive error following combined iStent or iStent injection implantation with cataract surgery is nearly equivalent to that following standalone cataract surgery [230]. However, it cannot be ruled out that the minimal IOP reduction achieved in these cases influenced the favorable outcomes.

In contrast to the iStent, AIT often achieves greater IOP reduction. Luebke et al. reported that the APE in 137 eyes that underwent TOM combined with cataract surgery was 0.53 ± 0.44 D. No significant difference was observed compared with the control group, which underwent cataract surgery alone, with 86.9% of eyes achieving a target refractive error within ±1.0 D [213]. However, in three eyes (2.2%), the refractive error exceeded ±2.0 D.

This study, published in 2015, used the SRK/T formula for IOL power calculation in 131 eyes with axial lengths between 21.0 and 26.0 mm, while the Haigis formula was used for six eyes with axial lengths > 26.0 mm. The authors suggested that using formulas that are less affected by axial length might have yielded different results.

Similarly, Sieck et al. reported that the target refractive error in eyes undergoing KDB combined with cataract surgery was within ±0.5 D in 73.7% of cases and within ±1.0 D in 93.4%, with no significant difference compared to cataract surgery alone [242]. In a multivariate analysis of risk factors for refractive errors exceeding ±1.0 D, KDB combined with cataract surgery was not identified as a risk factor (adjusted odds ratio: 0.5, 95% CI: 0.2–1.2, *p* = 0.32). However, a higher preoperative IOP was identified as a significant risk factor (adjusted odds ratio: 1.6, 95% CI: 0.8–3.5, *p* = 0.03). The authors speculated that axial length changes related to IOP reduction may have influenced these results, although postoperative axial length measurements were not included in the study. For GATT, which involves a 360° incision in the trabecular meshwork, Tekcan et al. analyzed 53 eyes that underwent GATT combined with cataract surgery. They reported significant postoperative axial length shortening; however, the APE using the Barrett Universal II formula was 0.36 ± 0.30 D [240]. In a comparative study of multiple procedures, Shaheen et al. evaluated 150 eyes that underwent angle incision surgery (84 GATT and 66 non-GATT), 395 eyes that underwent stent implantation (316 iStent and 79 Hydrus), and 7815 eyes that underwent cataract surgery alone. At 1 month postoperatively, the spherical equivalent was −0.36 ± 0.91 D for angle incision surgery, −0.31 ± 0.85 D for stent implantation, and −0.39 ± 0.88 D for standalone cataract surgery (*p* = 0.019). Although a slight statistical difference was observed between the groups, the authors concluded that this was unlikely to be clinically significant [253].

Most studies suggest that the impact of CB-MIGS combined with cataract surgery on postoperative target refractive error is minimal, with little difference compared with cataract surgery alone. However, Kanda et al. compared 52 eyes that underwent TMH combined with cataract surgery (TMH group) with 67 eyes that underwent cataract surgery alone (CAT group) [241]. At 1 month postoperatively, axial length was shortened by 0.16 mm in the TMH group and by 0.11 mm in the CAT group. The APE with the Barrett formula was 0.46 ± 0.42 D and 0.33 ± 0.24 D, respectively, while with the SRK/T formula, it was 0.62 ± 0.55 D and 0.42 ± 0.32 D, respectively. The APE was significantly larger in the TMH group compared with the CAT group (*p* = 0.04 for Barrett; *p* = 0.01 for SRK/T). For the TMH group, the APE with different IOL power calculation formulas was 0.46 ± 0.42 D for Barrett, 0.62 ± 0.55 D for SRK/T, and 0.76 ± 0.57 D for Haigis. The refractive predicted error (RPE) was 0.09 ± 0.62 D, 0.34 ± 0.76 D, and 0.65 ± 0.69 D, respectively, with Barrett yielding the lowest RPE. Compared with the CAT group, the TMH group showed a greater hyperopic refractive error, which the authors speculated could be influenced by changes in IOP and axial length. However, the magnitude of the refractive error was not significant enough to undermine the validity of TMH combined with cataract surgery. Considering the findings of Sieck et al. that a high preoperative IOP is a risk factor for postoperative refractive error [242], it may be advisable to select IOLs with a more myopic refractive target for patients with a high preoperative IOP, particularly when a significant reduction in IOP is anticipated. Furthermore, when performing CB-MIGS combined with cataract surgery, it is preferable to use IOL power calculation formulas that are less influenced by the corneal refractive power and axial length. The Universal II (Barrett) formula, which has been increasingly used in recent years, is less affected by axial length [254,255,256,257] and may be a suitable choice for CB-MIGS combined with cataract surgery. Moreover, eyes with extremely long axial lengths were often excluded from previous studies, as such eyes are known risk factors for the development and progression of glaucoma [258], frequently necessitating glaucoma surgery. Investigating the impact of IOP and axial length changes on the refractive error after CB-MIGS combined with cataract surgery is crucial and warrants further research.

### 7.1. Surgically Induced Astigmatism (SIA)

In filtration surgery, when cataract surgery is performed simultaneously, the incidence of postoperative refractive abnormalities and SIA is reportedly higher [237]. Additionally, SIA is induced in the direction of the surgical flap [259,260,261]. The possible mechanism of SIA after TLE may be tissue contraction around the TLE site secondary to extensive scleral cautery and suture, the removal of the second scleral flap, the wound-healing process of the subconjunctiva [261], and corneal steepening provoked by the pressure of a large drainage bleb under the eyelid [262]. Delbeke et al. reported that lower IOPs achieved after filtration surgery were associated with higher SIA and worse visual acuity [263].

In contrast, regarding exLOT, which requires conjunctival incisions and flap creation similar to TLE and CB-MIGS, which are performed using corneal incisions, Kanda et al. compared 52 eyes treated with TMH with 42 eyes treated with exLOT [96]. In the TMH, two corneal ports were created at the 1–2 o’clock, and 10–11 o’clock positions, and the trabecular meshwork was incised at 120–150°. At 1 week postoperatively, the SIA was 0.34 ± 0.67 diopters (D) (88 ± 18 degrees) in the TMH group and 0.28 ± 1.06 D (96 ± 20 degrees) in the exLOT group, with a significant difference observed between the two groups (*p* = 0.003). However, after 1 month, no significant differences were observed between the two groups, and at 3 months postoperatively, the residual SIA was minimal in both groups, measuring 0.06 ± 0.59 D (90 ± 17 degrees) in the TMH group and 0.07 ± 0.77 D (118 ± 18 degrees) in the exLOT group.

Although a scleral incision in exLOT results in unavoidable SIA during the first postoperative week, this is considered a temporary complication that resolves to a negligible level within 1 month. Similar findings were reported by Tanito et al. [264].

Numerous studies have reported the impact of CB-MIGS on SIA [241,251,252,265,266,267]. Regarding investigations involving the insertion of two iStent inject devices, Ioannidis et al. reported that in 106 eyes that underwent combined cataract surgery, no postoperative worsening of astigmatism was observed, and 73.8% of cases had residual refractive astigmatism of ≤0.5 D [252]. Rho et al. compared combined cataract surgery with cataract surgery alone and found no significant differences in SIA between the two groups [251].

Regarding AIT, Kanda et al. reported that in 52 eyes that underwent TMH combined with cataract surgery, the SIA was 0.22 ± 0.85 D (78 ± 19 degrees), which was not significantly different from the SIA in the control group of 67 eyes that underwent only cataract surgery [241]. Similarly, Hirabayashi et al. reported comparable findings in a study using the KDB [265].

Regarding SIA after ABiC, Byszewska et al. examined 37 eyes that underwent ABiC combined with cataract surgery. They reported that the centroid of astigmatism (mean cylinder with axis) was 0.79 D (172° ± 1.10) preoperatively, 0.75 D (166° ± 1.01) at 6 months postoperatively, and 0.64 D (164° ± 1.11) at 24 months postoperatively. No visually significant astigmatism was observed, the mean direction of astigmatism remained stable, and the degree of astigmatism showed no significant changes throughout the 24-month observation period [266].

Furthermore, Wilczynski et al. investigated the differences in SIA caused by different cataract surgical techniques. They compared 58 eyes in which IOLs were implanted through a 1.8 mm temporal corneal incision with coaxial CB-MIGS (coaxial group) and 50 eyes in which IOLs were implanted using a bimanual technique through a 1.7 mm corneal incision and a 1.5 mm side port for an irrigating chopper, followed by CB-MIGS (bimanual group). Based on vector analysis, the SIA was 0.42 ± 0.29 in the coaxial group and 0.50 ± 0.24 in the bimanual group, with no significant difference between the two groups (*p* > 0.05) [267].

Thus, because CB-MIGS is performed using small corneal incisions, SIA caused by the corneal incisions is minimal. Even in combined surgeries, accounting for additional SIA from CB-MIGS beyond the SIA associated with cataract surgery is considered unnecessary [242,265].

### 7.2. Using Toric IOLs During CB-MIGS Combined with Cataract Surgery

Tanito et al. compared the SIA at 3 months postoperatively across four surgical procedures, TLE, EX-PRESS^®^ shunt (EXP), exLOT, and TMH, with 20 eyes analyzed for each procedure. The reported SIA values were as follows: 1.01 ± 2.27D (56 ± 26°) for TLE, 0.62 ± 1.20D (74 ± 24°) for EXP, 0.23 ± 1.04D (112 ± 17°) for exLOT, and 0.12 ± 0.75D (97 ± 21°) for TMH. TLE showed a significantly greater SIA than other procedures (*p* = 0.0001) [265].

Additionally, TLE exhibited significant variability in SIA between cases, making it nearly impossible to predict SIA. Consequently, toric IOLs are rarely used in combined cataract surgery. In contrast, for CB-MIGS, accounting for SIA from the procedure itself, beyond the SIA caused by cataract surgery, is unnecessary. Therefore, the use of toric IOLs during CB-MIGS combined with cataract surgery should be considered in cases of pre-existing regular corneal astigmatism.

Ichioka et al. investigated the outcomes of toric IOL implantation in eyes with pre-existing regular corneal astigmatism > 1.5D. They compared nine eyes (nine patients) implanted with toric IOLs during combined cataract surgery with an iStent (toric group) and nine eyes (nine patients) implanted with non-toric IOLs (non-toric group) [268]. In the toric group, uncorrected visual acuity (UCVA) improved to −0.21 ± 0.06 (logMAR) (*p* = 0.02), and refractive astigmatism improved to +1.72 ± 0.25D (*p* = 0.0039). Furthermore, 78% of eyes in the toric group achieved a refractive astigmatism of –1.0 D, compared with only 11% in the non-toric group.

Similarly, Takai et al., from the same group, reported on 10 eyes (10 patients) with pre-existing regular corneal astigmatism > 1.5 D. They compared eyes implanted with toric IOLs during combined cataract surgery with TMH (toric group) with eyes implanted with non-toric IOLs (non-toric group) [269]. At 3 months postoperatively, the mean UCVA in the toric group was 0.23 ± 0.25 (logMAR), significantly better than 0.45 ± 0.26 (logMAR) in the non-toric group (*p* < 0.05). The mean absolute residual refractive cylinder in the toric group was 1.30 ± 0.68 D, which was significantly smaller than 2.25 ± 0.62 D in the non-toric group (*p* < 0.05). However, SIA was 0.77 ± 0.43 D in the toric group and 0.60 ± 0.32 D in the non-toric group, with no significant difference observed. Based on these results, the authors concluded that while toric IOLs improve outcomes compared with non-toric IOLs, some residual astigmatism remains.

Ichioka et al. reported similar findings under comparable conditions [270]. Their results slightly favored the toric group compared with those of Takai et al., suggesting that toric IOL implantation may lead to better visual outcomes in eyes with pre-existing regular corneal astigmatism.

SIA includes not only corneal astigmatism but also factors such as toric IOL rotation, tilt, or decentration of the lens capsule or IOL, and unknown ocular components [271]. In eyes with high myopia and loose capsules or in those with zonular weakness associated with XFG, the IOL and capsule tend to rotate and tilt [271,272]. In Takai et al.’s study of the 10 patients in the toric group, two patients with high myopia and XFG exhibited a slight worsening of absolute refractive astigmatism postoperatively compared to preoperative values. Although postoperative toric IOL rotation was not assessed, making the details unclear, the authors speculated that IOL rotation or tilt may have occurred in these two cases.

Whether the procedural steps involved in CB-MIGS or the reduction in IOP contribute to toric IOL rotation or tilt remains uncertain, as there is currently insufficient research on this topic. Further studies involving larger numbers of patients are required to clarify this issue. However, based on previous reports, the use of toric IOLs during CB-MIGS combined with cataract surgery appears to be a reasonable option for patients with significant corneal astigmatism.

In contrast, in patients with high myopia or XFG—conditions that are common indications for glaucoma surgery—preoperative astigmatism may not be effectively corrected. Moreover, if these patients undergo TLE, the resulting optical aberrations could negatively affect their vision [269]. Therefore, the decision to use toric IOLs in such cases requires the careful consideration of factors such as patient age, disease stage, and disease type.

## 8. Effectiveness in Slowing the Progression of Visual Field Defects

Numerous studies have reported that the postoperative progression of visual field defects can be significantly delayed in filtration surgery [5,273,274,275,276]. In contrast, Bao et al. conducted a long-term study with an average follow-up period of 8 years (with a minimum of 6 years of observation) and reported that exLOT was inferior to TLE regarding both IOP control and effectiveness in slowing the progression of visual field defects [277].

Several studies have examined the effect of CB-MIGS in slowing the progression of visual field defects. These studies consistently reported that the progression rate after CB-MIGS was slow; however, most were short term and retrospective [92,193,278,279]. Kitamura et al. conducted a relatively long-term study evaluating the progression of visual field defects over 4 years after trabeculotomy using TOM in 250 eyes (POAG, 63%; XFG, 16%; others, 21%) [53].

Using the Humphrey visual field analyzer SITA-standard 24-2 program (HFA24-2), they reported that the mean deviation (MD) values were 10.5 ± 7.0 dB preoperatively and 11.4 ± 7.8 dB at 4 years postoperatively, with no significant worsening observed at any observation point compared to preoperative values. However, 62% of the patients underwent simultaneous cataract surgery, and the number of cases observed after the third postoperative year was less than half of the initial sample size, which requires a careful interpretation of the results.

Weber et al. reported on 264 eyes (POAG: 64%; XFG: 20%; others: 16%) with a mean follow-up period of 45.3 months after trabeculotomy using the TOM [52]. The rate of visual field progression remained relatively stable over 5 years, with MD values measured using the HFA24-2 reported as −11.1 dB preoperatively and −11.0 dB at 4 years postoperatively, showing no significant worsening.

Notably, this study maintained a relatively large sample size for long-term follow-up compared with other reports, with 142 eyes analyzed at 3 years postoperatively, 97 eyes at 4 years, and 68 eyes at 5 years. However, 74 patients (28.0%) required additional glaucoma surgery at a mean of 18.3 months postoperatively; these patients were excluded from further analysis, introducing a potential selection bias.

In addition, 40% of the patients underwent TOM alone, whereas 60% underwent combined cataract surgery. At all time points during the follow-up period, no significant differences in the MD values were observed between the two groups, except at the final five-year follow-up. At this time point, the MD value in the combined cataract surgery group (−10.35 dB) was significantly better than in the TOM-alone group (−12.04 dB) (*p* = 0.016).

Regarding KDB, Vasu et al. examined 90 eyes and reported MD values of −3.56 dB preoperatively and −5.32 dB at 6 years postoperatively using HFA24-2 [280]. Although a statistically significant difference was observed (*p* = 0.02), the progression rate was not considered to be clinically rapid.

Regarding ABiC, Koerber et al. reported on 27 eyes treated with the iTrack microcatheter, evaluating visual field changes over a maximum of 4 years [157]. The MD values using HFA24-2 were −7.86 ± 6.90 dB preoperatively and −6.95 ± 5.72 dB postoperatively, with no significant worsening observed (*p* = 0.634). However, caution is warranted when interpreting these results because of unclear details regarding the final observation points and the inclusion of cases that underwent combined cataract surgery.

In contrast, prospective randomized controlled trials (RCTs) have reported changes in the visual field after Hydrus device implantation. Montesano et al. conducted a multicenter RCT comparing a group of 352 eyes that underwent combined Hydrus and cataract surgery (Hydrus group) with a group of 165 eyes that underwent cataract surgery alone (CAT group). The study used 5-year visual field data collected during the prospective, randomized, multicenter HORIZON trial. The MD slope > 5 years in the Hydrus group was −0.26 dB/year, which was significantly slower than −0.49 dB/year in the CAT group (95% confidence interval [CI]: −0.63, −0.34; *p* = 0.014) [281].

However, differences in mean daytime IOP control accounted for only 17% of the difference in progression rates between the groups (−0.04 dB/year, 95% CI: −0.06, −0.02; *p* < 0.0001), leaving a large and significant unexplained difference in progression rates (–0.19 dB/year, 95% CI: −0.37, −0.02; *p* = 0.0328, comprising 83% of the total difference).

One potential explanation for this residual difference is that the reduction in diurnal IOP fluctuations achieved by the device placement may have contributed to the slower progression rate. However, this hypothesis needs to be confirmed through 24-hour IOP monitoring [282].

In another report on micro-implantable devices, Gillmann et al. conducted a meta-analysis of 1115 eyes that underwent iStent surgery. The weighted mean follow-up period was 37.9 months (range: 12–96 months), and the baseline weighted mean IOP was 19.0 ± 3.1 mmHg, which showed a mean reduction of 26.6% at the end of follow-up (range: 15.2–42.3%). The weighted mean MD values during the follow-up period changed from −5.76 ± 5.68 dB to −5.91 ± 5.82 dB, with an overall MD slope (MDS) of −0.024 ± 0.34 dB/year. In the cataract-combined surgery group, the MDS was −0.01 ± 0.42 dB/year, while the iStent-alone group was −0.07 ± 0.62 dB/year [283].

This rate of progression is comparable to that reported in normal, non-glaucomatous eyes [284,285] and slower than that reported in glaucomatous eyes treated with medications [284,285,286,287,288], making it a remarkable finding. Gillmann et al. hypothesized that this result may be attributed to a reduction in IOP fluctuations achieved by surgery and improved patient compliance due to early surgical intervention.

However, issues such as the reliability of visual field test results, the impact of disease severity, and the exclusion of cases requiring additional glaucoma surgery introduce several unavoidable biases. Whether device implantation has a greater effect in slowing visual field progression than incisional procedures remains unclear owing to the lack of reliable comparative studies. Although a definitive interpretation cannot be made yet, future research is needed to clarify whether placing a stent to maintain a stable, enclosed space within Schlemm’s canal is superior to incising and opening the canal or whether differences in diurnal IOP fluctuations between the two techniques play a role.

Regarding differences in visual field preservation based on disease type, Weber et al. reported 168 eyes with POAG and 52 eyes with XFG in a study with a mean follow-up of 45.3 months after TOM. They found no significant difference in the rate of visual field progression between the two groups during the observation period [52]. However, a separate report investigating the rate of visual field progression over 5.8 years in untreated cases found that XFG progressed at a rate of −1.13 dB/year, which was faster than the rates for normal-tension glaucoma (−0.22 dB/year) and POAG (−0.46 dB/year) [289]. These findings suggest that XFG requires careful monitoring as a high-risk disease.

In our analysis, the cumulative survival rate for 63 eyes with XFG treated with TOM was relatively favorable during the first 2–3 years postoperatively but showed a sharp decline after 4 years. While some cases involve IOP elevation, many others experience the progression of visual field defects or the deterioration of visual acuity despite relatively good IOP control, necessitating additional filtration surgery and ultimately being deemed treatment failures [18].

As a limitation of LOT-type surgeries for XFG, Honjo et al. showed that visual field defects may progress even when IOP is well controlled after exLOT [290], and Konstas et al. suggested that factors other than IOP may contribute to disease progression [291]. For XFG, even when IOP is relatively well controlled following CB-MIGS, a close follow-up with scheduled visual field testing at shorter intervals is necessary to carefully monitor disease progression.

## 9. Conclusions

Numerous studies have shown that CB-MIGS has a strong safety profile, with a low incidence of complications leading to severe visual impairment. Additionally, many studies support its effectiveness in preserving visual acuity and slowing the progression of visual field defects. However, recent reports have raised concerns about its long-term efficacy. In advanced cases where preoperative sensitivity near the fixation point has already deteriorated, there is a risk of irreversible postoperative vision loss, highlighting the need for careful patient selection. While some studies have conducted relatively long follow-ups, most are retrospective, and in many cases, fewer than half of the preoperative cohort have data available beyond three to four years postoperatively. Given the current research, it remains difficult to definitively confirm that CB-MIGS provides sustained benefits in preserving visual acuity and slowing visual field progression. Moving forward, long-term prognostic reports from prospective randomized controlled trials (RCTs) are urgently needed.

## Figures and Tables

**Table 1:** Summary of Principal Complications (%) Leading to Visual Decline by Device Type.

Surgical Technique	Hyphema (%)	IOP Spikes (%)	Corneal Edema (%)	CME (%)	Severe Complications (e.g., Endophthalmitis, Suprachoroidal Hemorrhage) (%)
iStent/iStent inject	1.1–22.2	0–11.4	0–10.0	1.0–1.3	Not specified
Hydrus	1.9–6.5	1.9–6.5	0–3.2	2.0	Not specified
AIT (KDB, TOM, TMH)	0–95.0	1.0–28.9	0–17.2	1.8–7.0	0.01% (Endophthalmitis)
GATT	2.0–100.0	0–49.0	0–14.7	0–6.2	0–1.1% (Suprachoroidal Hemorrhage)
ABiC	1.0–47.0	0–42.0	0–4.9	0–4.9	Not specified

IOP = intraocular pressure, CME = Cystoid macular edema, AIT = ab interno trabeculotomy, KDB = Kahook Dual Blade, TOM = Trabectome, TMH = Tanito Microhook, GATT = gonioscopy-assisted transluminal trabeculotomy, ABiC = ab interno canaloplasty.

**Table 2:** Summary of Refractive Changes After CB-MIGS Combined with Cataract Surgery.

Study	Procedure	Sample Size	APE (D)	% Within ±0.50 D	% Within ±1.0 D	Significant Difference vs. Standalone Cataract Surgery
Scott et al. [250]	iStent	76	Not specified	80%	95%	No
Rho et al. [251]	iStent Inject	36	0.33 ± 0.26	83.3%	97.2%	No
Ioannidis et al. [252]	iStent Inject	106	0.36 ± 0.25	73.9%	98.9%	No
Luebke et al. [213]	TOM	137	0.53 ± 0.44	60.6%	86.9%	No
Sieck et al. [242]	KDB	76	Not specified	73.7%	93.4%	No
Tekcan et al. [240]	GATT	53	0.38 ± 0.32 (SRK/T) 0.36 ± 0.30 (Barrett) 0.32 ± 0.28 (Hill-RBF) 0.30 ± 0.28 (Kane)	66.0% (SRK/T) 69.8% (Barrett) 73.6% (Hill-RBF) 79.2% (Kane)	100.0%	Not Compared
Kanda et al. [241]	TMH	52	0.46 ± 0.42 (Barrett) 0.62 ± 0.55 (SRK/T) 0.76 ± 0.57 (Haigis)	63.5% (Barrett) 57.7% (SRK/T) 38.5% (Haigis)	Not specified	Yes (P = 0.04 for Barrett, P = 0.01 for SRK/T)

APE = Absolute Prediction Error, TOM = Trabectome, KDB = Kahook Dual Blade, GATT = gonioscopy-assisted transluminal trabeculotomy, TMH = Tanito Microhook, SRK/T = Sanders-Retzlaff-Kraft/theoretical, Barrett = Barrett Universal ΙΙ, Hill-RBF: Hill-radial basis function.

## Abbreviations

The following abbreviations are used in this manuscript:

ABiC	Ab interno canaloplasty
ACD	Anterior chamber depth
AIT	Ab interno trabeculotomy
APE	Absolute predicted error
BCVA	Best-corrected visual acuity
CAT	Cataract surgery alone
CB-MIGS	Canal-based minimally invasive glaucoma surgery
CI	Confidence interval
CME	Cystic macular edema
ELP	Effective lens position
ExLOT	Trabeculotomy ab externo
GATT	Grover introduced gonioscopy-assisted transluminal trabeculotomy
IOL	Intraocular lens
IOP	Intraocular pressure
KDB	Kahook dual blade
MD	Mean deviation
MIGS	Minimally invasive glaucoma surgery
POAG	Primary open-angle glaucoma
RCT	Randomized controlled trial
RPE	Refractive predicted error
SIA	Surgically induced astigmatism
TLE	Trabeculectomy
TMH	Tanito Microhook
TOM	Trabectome
UCVA	Uncorrected visual acuity
XFG	Exfoliation glaucoma
